# Lamellar Septa-like Structured Carbonate Apatite Scaffolds with Layer-by-Layer Fracture Behavior for Bone Regeneration

**DOI:** 10.3390/biomimetics9020112

**Published:** 2024-02-14

**Authors:** Ahmad Nazir Taleb Alashkar, Koichiro Hayashi, Kunio Ishikawa

**Affiliations:** Department of Biomaterials, Faculty of Dental Science, Kyushu University, 3-1-1 Maidashi, Higashi-ku, Fukuoka 812-8582, Japan

**Keywords:** biomimetics, bioinspired materials, cuttlebone, scaffold, tissue engineering

## Abstract

Generally, ceramics are brittle, and porosity is inversely correlated with strength, which is one of the challenges of ceramic scaffolds. Here, we demonstrate that lamellar septum-like carbonate apatite scaffolds have the potential to overcome these challenges. They were fabricated by exploiting the cellular structure of the cuttlebone, removing the organic components from the cuttlebone, and performing hydrothermal treatment. Scanning electron microscopy revealed that the scaffolds had a cellular structure with walls between lamellar septa. The interwall and interseptal sizes were 80–180 and 300–500 μm, respectively. The size of the region enclosed by the walls and septa coincided with the macropore size detected by mercury intrusion porosimetry. Although the scaffold porosity was extremely high (93.2%), the scaffold could be handled without disintegration. The compressive stress–strain curve demonstrated that the scaffolds showed layer-by-layer fracture behavior, which seemed beneficial for avoiding catastrophic failure under impact. When the scaffolds were implanted into rabbit femurs, new bone and blood vessels formed within the scaffold cells at 4 weeks. At 12 weeks, the scaffolds were almost entirely replaced with new bone. Thus, the lamellar septum-like cellular-structured carbonate apatite is a promising scaffold for achieving early bone regeneration and compression resistance.

## 1. Introduction

Bone defects are caused by trauma due to falls, tumbling, collisions, and car accidents. Furthermore, these are often caused by aging and certain diseases (Alzheimer’s and Parkinson’s) [1,2,3,4]. The treatment of these defects, particularly critically sized defects, primarily involves the use of bone grafts. Autografts are considered the gold standard of bone graft materials because of their biocompatibility, bioresorbability, and osteoconductivity [1,2,3]. However, they have some limitations, including the limited availability of harvested bone and the need for additional surgical procedures, which include complications such as site morbidity, extended healing time, and prolonged hospitalization [4,5,6,7,8]. This has led to a growing interest in exploring synthetic bone grafts to overcome these drawbacks.

Primary studies have focused on the chemical composition of synthetic bone grafts and scaffolds. Calcium phosphates are conventionally used because they are similar in composition to the inorganic components of human bone [8,9,10,11,12,13,14,15,16]. Although the scaffold composition determines the presence or absence of osteoconductivity, the scaffold structure provides most of the important characteristics for bone regeneration, such as mechanical strength, cell migration, ingrowth of bone and blood vessels, protein adsorption, and nutrient permeation. Furthermore, the scaffold structure affects the removal of waste products resulting from cellular metabolism because it is involved in the distribution of body fluids and blood. Thus, waste products are easily removed when the scaffold structure is more conducive to body fluid and blood distribution. This prevents waste products from reaching toxic levels and provides a favorable environment for viable cells to form bones [17,18,19,20,21]. The porosity and interconnectivity of scaffolds conventionally control these characteristics. Generally, the higher the porosity of a scaffold, the lower its strength.

To overcome this, scaffold structures have been designed to mimic living organisms. The skeleton of cuttlefish, a marine mollusk of the order *Sepiida*, known as cuttlebone (CB), is an example of a unique biological structure [22]. The cellular structure of CB is composed of lamellar septa separated by asymmetric, distorted, and S-shaped walls. Although CB exhibits extremely high porosity (approximately 90%), it also exhibits layer-by-layer failure behavior owing to its lamellar septum-like structure [23,24]. This fracture behavior may help prevent the entire scaffold from immediately breaking when impacted. This attribute could be particularly advantageous for ceramic scaffolds because ceramics are inherently fragile and usually experience a complete break when subjected to a load that exceeds their breaking loads. Notably, even octet-truss lattices, Kelvin foams, and gyroid structures, which are mechanically superior porous structures, do not exhibit layer-by-layer failures [25]. Therefore, the structural properties of CB can alleviate the inherent weaknesses of ceramic scaffolds.

Notably, more than 90% of CB is composed of CaCO_3_, which can be used as a calcium phosphate precursor [22,26]. Therefore, previous studies have focused on the chemical composition of CB to prepare hydroxyapatite (HAp) and reduce the waste generated by the food industry [27,28]. However, because most of these studies did not focus on the fracture behavior of CB, they produced powdered HAp that lacked the unique mechanical characteristics of CB [29,30,31]; even though they fabricated block-type HAp, they did not evaluate the mechanical properties of the products [32]. Furthermore, few studies have evaluated the in vivo bone-formation ability of products synthesized from CB; therefore, their usefulness as scaffolds for bone regeneration is not well understood.

Scaffolds that are gradually resorbed after bone formation are favorable for bone regeneration. Some researchers have reported that a small amount of HAp is resorbed and remains there for several years after implantation. Beta-tricalcium phosphate (β-TCP) spontaneously dissolves before bone formation, resulting in a loss of function as a scaffold for cells [33,34,35,36]. In contrast, carbonate apatite (CAp) is gradually resorbed and replaced by new bone [37,38,39]. Therefore, CAp might be more suitable than HAp as a scaffold for bone regeneration. However, CAp scaffolds have not been fabricated using CB. Hence, this study aimed to establish a method for fabricating lamellar septum-like structured CAp scaffolds from CB (CAp-CB scaffolds) and to elucidate their mechanical properties and in vivo bone-formation ability.

## 2. Materials and Methods

### 2.1. Preparation of CAp-CB Scaffolds

Cleaned and dried CB derived from *Sepia esculenta* was purchased from NICHIDO, Inc. (Tokyo, Japan) and used as received. The CAp-CB scaffolds were prepared as follows. First, the rigid dorsal shells of the cuttlefish were removed to facilitate further processing. A trephine drill with an inner diameter of 6 mm was used to obtain CB cylinders with a height of approximately 4 mm and a diameter of approximately 6 mm. To eliminate organic components, raw CB cylinders (approximately 2 g) were immersed in a 12% sodium hypochlorite (NaClO) solution (50 mL, Honmachi Chemical Industry Co., Ltd., Tokyo, Japan). The air included in the CB cylinders was removed under vacuum to facilitate the permeation of the NaClO solution into the entire CB cylinder. The CB cylinder was immersed in the NaClO solution at 20 °C for 24 h in the dark. Then, the CB cylinders were collected from the NaClO solution and thoroughly rinsed with distilled water, followed by immersion in distilled water at 80 °C overnight. The washed CB cylinders were dried at 120 °C for 8 h in a thermostatic oven (VTRF-27; ISUZU CAP, Niigata, Japan). After removal of organic components, the CB cylinders were subjected to hydrothermal treatment (HT) in a 1 mol/L disodium hydrogen phosphate (Na_2_HPO_4_) aqueous solution using a TEFLON-lined stainless steel pressure vessel at 120 °C for 7 d. Thus, CAp-CB scaffolds were obtained. The vessels were allowed to cool down and then the CAp-CB scaffolds were washed with 80 °C distilled water and dried at 100 °C. The CAp-CB scaffolds underwent dry-heat sterilization at 170 °C for 3 h for animal experiments.

### 2.2. Characterization of CAp-CB Scaffolds

#### 2.2.1. Structural Analysis

Gross and microcomputed tomography (μ-CT) images of the raw and NaClO-treated CB cylinders and CAp-CB scaffolds were acquired using a digital microscope camera (Leica MC170 HD 5 MP HD; Leica Microsystems GmbH, Wetzlar, Germany) and μ-CT scanner (Skyscan 1075 KHS; Kontich, Belgium). The scanning parameters were as follows: resolution 6.8 μm; voltage 60 kV; and current 120 µA. The acquired CT slice images were reconstructed using CTAn software 5.30 (CT Analyzer, Bruker, Belgium). The microstructures of the raw and NaClO-treated CB cylinders and CAp-CB scaffolds were examined using scanning electron microscopy (SEM; S-3400 N; Hitachi High Technologies Co., Tokyo, Japan) at a voltage of 10 kV after coating the cylinders and scaffolds with a gold–palladium alloy through magnetron sputtering. 

#### 2.2.2. Organic Residue Evaluation

Thermogravimetric differential thermal analysis (TG-DTA) of the raw and NaClO-treated CB cylinders was conducted to investigate the removal of organic materials. These CB cylinders were heated to 850 °C at a heating rate of 0.5 °C/min under air for TG-DTA measurements. The data were obtained at 180 s intervals.

#### 2.2.3. Composition Analysis

The composition of the raw and NaClO-treated CB cylinders and CAp-CB scaffolds was determined by X-ray diffraction (XRD; D8 Advance; Bruker AXS GmbH, Karlsruhe, Germany), and Fourier-transform infrared spectroscopy (FT-IR; FT/IR-6200; JASCO, Tokyo, Japan) for CAp-CB scaffolds was conducted. The CAp-CB scaffolds were ground into a fine powder and dried before the FT-IR measurements. The reference XRD pattern and FTIR spectrum of CAp were obtained using commercial CAp granules (Cytrans granules; GC, Tokyo, Japan).

#### 2.2.4. Carbonate Content Analysis

The carbonate content of the CAp-CB scaffolds was analyzed using a carbon–hydrogen–nitrogen (CHN) Corder (MT-5; Yanako, Tokyo, Japan). The average carbonate content was calculated from the results of five samples.

#### 2.2.5. Bulk Porosity

The bulk porosity of raw and NaClO-treated CB cylinders and CAp-CB scaffolds was calculated using the following formula: BP = {1 − (TD/D)} × 100, where BP is the bulk porosity, D is the density of each sample determined by dividing the weight by the volume, and TD is the theoretical density. The aragonite, hydroxyapatite, and raw CB densities were 2.95, 3.05, and 2.83 g/cm^3^, respectively. The average bulk porosity was measured using five samples per group. 

#### 2.2.6. Pore Size Distribution and Volume Analysis

The size distribution and volume of open pores in the raw and NaClO-treated CB cylinders and CAp-CB scaffolds were determined using mercury injection porosimetry (MIP; AutoPore 9420; Shimadzu, Kyoto, Japan).

#### 2.2.7. Compressive Strength Test

The compressive strengths of raw CB cylinders and CAp-CB scaffolds were measured using a universal testing machine (Autograph AGS-J; Shimadzu, Kyoto, Japan). Eight samples were used for each group. The compression direction was perpendicular to that of the CB layer, and the compression rate was 0.5 mm/min.

### 2.3. Animal Experiments

The animal protocol used in this study was approved by the Animal Care and Use Committee of Kyushu University (No. 22-086-0, issued on 17 February 2022). Eight Japanese white rabbits (male, 18–19 weeks old, weighing 3.0–3.5 kg) from Japan SLC (Hamamatsu, Japan) were acquired. The surgical procedure was as follows: after anesthetizing the animals, the fur covering the femur area was shaved, and the skin was disinfected. 

A bone defect that is 6 mm in diameter and 4 mm in depth was created in the distal epiphysis of both rabbit femurs by drilling using a trephine bur (6 mm in outer diameter). The CAp-CB scaffold was implanted into the bone defect of one leg, and no scaffold was implanted in bone defect of other leg (empty group), which was used as negative control. Femurs were collected 4 and 12 weeks after surgery and preserved in a 10% formalin-neutral buffer solution. Four legs were assigned to each treatment and empty group (*n* = 4 per group).

### 2.4. µ-CT and Histological Evaluation

Rabbit femurs collected at 4 and 12 weeks after surgery were fixed in a 10% formalin solution. µ-CT images of the femurs were obtained by µ-CT scanning (Skyscan 1075 KHS). The scanning parameters were as follows: resolution 24 μm; voltage 60 kV; and current 120 µA. Using standard methods, fixed rabbit femurs were embedded in paraffin blocks, 3 μm tissue sections were prepared by slicing the paraffin blocks in a direction perpendicular to the CB layer of the scaffold, and hematoxylin–eosin (HE) staining of tissue sections was performed. The percentage of new bone area was calculated using the formula NB/TA × 100, where NB and TA are the new bone area and total area of the bone defect, respectively. 

### 2.5. Statistical Analysis

Sample size calculations were performed using PS Power and Sample Size Calculation software (version 3.1.6, released in October 2018 by William D. Dupont and Walton D. Plummer, Jr., Vanderbilt University, Nashville, TN, USA), where *p*-value < 0.05, the ratio between the groups was set to 1, and the desired power was 0.8. The differences between the means and standard deviations of the groups were set to 25 and 10, respectively. The calculations revealed that the required sample size was four per group (*n* = 4). 

All quantitative data are presented as the mean ± standard deviation. Statistical analysis was performed using Student’s *t*-test and a one-way ANOVA test using the Prism 9.5 (GraphPad Software Inc., La Jolla, CA, USA) software. Statistical significance was set at *p* < 0.05.

## 3. Results and Discussion

### 3.1. Structural and Physicochemical Properties of CAp-CB Scaffolds

**Figure 1:**
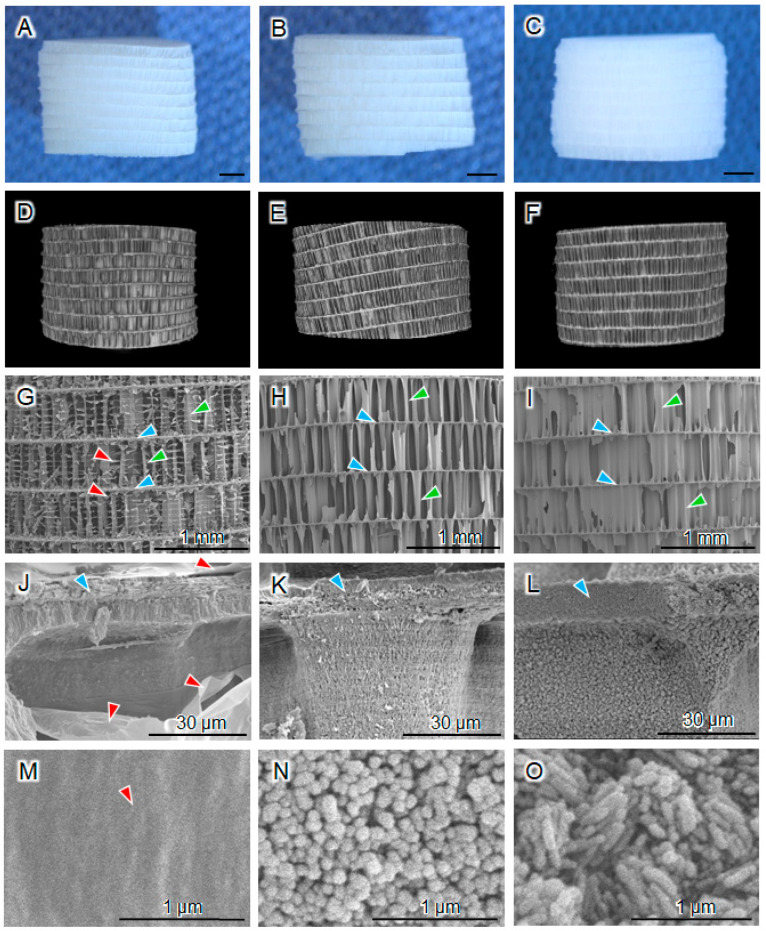
(**A**–**C**) Gross images, (**D**–**F**) µ-CT images, (**G**–**L**) SEM images, and (**M**–**O**) high-magnification SEM images of raw CB, NaClO-treated CB, and CAp-CB scaffolds. Scale bars: (**A**–**I**) 1 mm, (**J**–**L**) 30 µm, and (**M**–**O**) 1 µm. The green, blue, and red arrowheads indicate the walls, lamellar septa, and organic layer, respectively.

The shapes of the raw CB cylinders (Figure 1A) were maintained in the NaClO-treated CB cylinders (Figure 1B) and CAp-CB scaffolds (Figure 1C). The μ-CT images showed that all raw CB cylinders (Figure 1D), NaClO-treated CB cylinders (Figure 1E), and CAp-CB scaffolds (Figure 1F) possessed a cellular structure with walls between the lamellar septa. The SEM images showed that although the raw CB cylinders possessed a lamellar septum-like cellular structure, organic membranes were present between the walls of the raw CB cylinders (Figure 1G). The organic membranes disappeared in the NaClO-treated CB cylinders, while maintaining a lamellar septum-like cellular structure (Figure 1H). Thus, the NaClO treatment effectively removed the organic components from the raw CB cylinders while maintaining the lamellar septum-like cellular structure. The lamellar septum-like cellular structure was maintained in the CAp-CB scaffolds, indicating that HT did not destroy this structure (Figure 1I). High-magnification SEM images revealed that the smooth surface of raw CB (Figure 1J) changed to a rough surface for the NaClO-treated CB cylinders (Figure 1K), indicating that the organics in raw CB were removed and aragonite crystals appeared. The surfaces of the CAp-CB scaffolds were rougher than those of the NaClO-treated CB cylinders (Figure 1L), suggesting that the crystals grew during hydrothermal treatment. Higher magnification SEM images elucidated the changes in the crystal morphology from spherical—a characteristic of CaCO_3_ crystals—to rod-shaped (a characteristic of CAp) (Figure 1M–O). 

**Figure 2:**
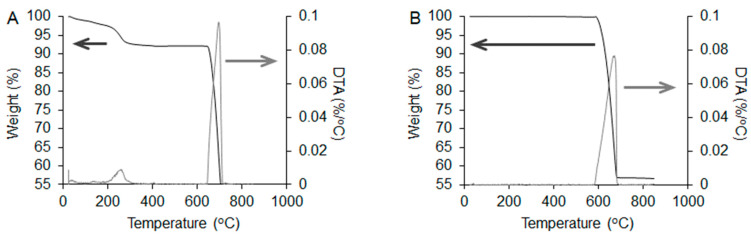
TG-DTA curves of (**A**) raw and (**B**) NaClO-treated CB cylinders. The dark and light gray arrows indicate the values of TG and DTA, respectively.

The effectiveness of the NaClO treatment in removing organic components from raw CB was evaluated by TG-DTA of raw CB and NaClO-treated CB cylinders (Figure 2). The DTA curve of raw CB cylinders showed two peaks at 260 °C and 690 °C, which coincided with the decomposition temperature of organic materials in raw CB and CaCO_3_, respectively (Figure 2A) [40]. In the TG curve, approximately 6% of weight loss was observed at 600 °C, which coincides with the organic content of raw CB (Figure 2A) [40]. In contrast, no organic-related weight loss or exothermic peaks were detected in the TG or DTA curves of the NaClO-treated CB cylinders (Figure 2B). Thus, the SEM and TG-DTA results indicate that the NaClO treatment was sufficient to remove organic components from raw CB. Furthermore, in the TG curve of NaClO-treated CB cylinders, the weight loss starting at approximately 600 °C was caused by the decomposition of CaCO_3_ to CaO (Figure 2B).

Conventionally, organics in the CB are removed through heating treatment above 600 °C [28,30,31]. However, conventional methods can affect the structural integrity of CB because of the thermal decomposition of CaCO_3_ into CaO. Furthermore, sintering decreases the number of micro/nanopores because of the fusion between neighboring crystals. Therefore, diffusion of the phosphate solution into the CaCO_3_ material becomes more difficult as the sintering temperature increases. Consequently, CaCO_3_ remains, and pure CAp cannot be easily obtained. Moreover, crystallinity increases with increasing sintering temperatures. The increased crystallinity also delays the conversion of CaCO_3_ into CAp. To completely convert CaCO_3_ materials with high crystallinity and a small portion of micro/nanopores into CAp, phosphatization by hydrothermal treatment at higher temperatures for longer periods is required. Higher temperatures and longer hydrothermal treatment durations increase the crystallinity of CAp and decrease its carbonate content. The resulting CAp is considered to be closer to HAp and different from the crystallinity and carbonate content of natural bones.

**Figure 3:**
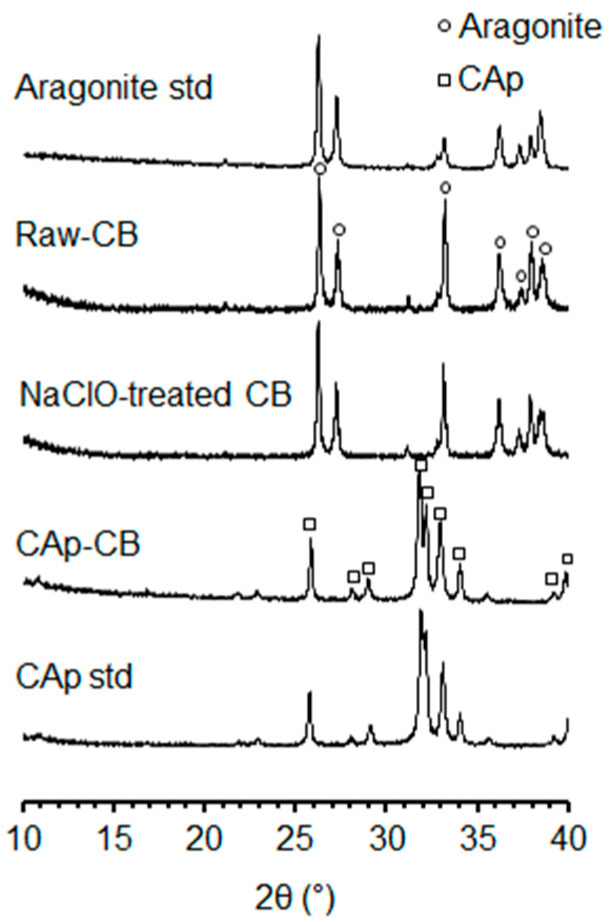
XRD patterns of the aragonite standard (std), raw and NaClO-treated CB cylinders, CAp-CB scaffold, and CAp standard (std).

The XRD patterns show that the raw and NaClO-treated CB cylinders consisted of CaCO_3_ (aragonite) (Figure 3). Thus, the NaClO treatment did not affect the crystal phase of CB. The diffraction pattern of the CAp-CB scaffolds coincided with that of the CAp standard, indicating that aragonite was converted to CAp through HT at 120 °C for 7 d.

**Figure 4:**
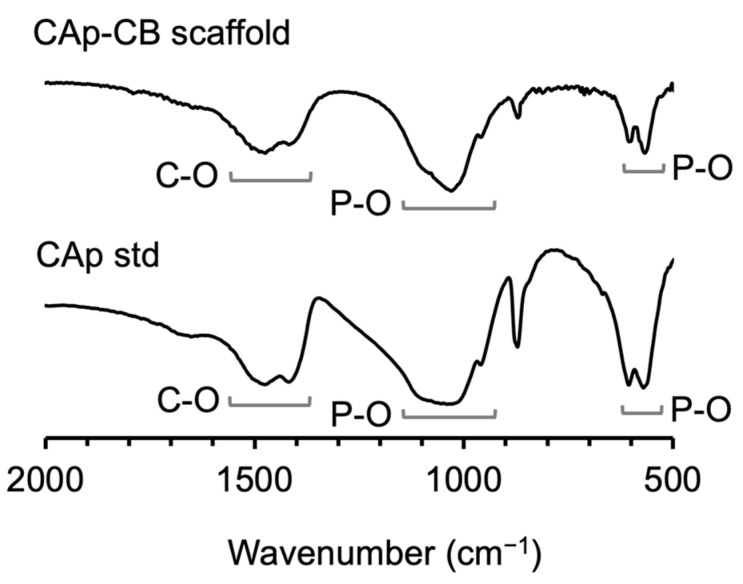
FT-IR spectra of CAp-CB scaffolds and CAp std.

The FT-IR spectra showed both carbonate and phosphate groups. The carbonate groups were detected in both the A and B sites (1539 and 1364 cm^−1^, respectively), representing the replacement of the hydroxyl (OH^−^) and phosphoric (PO_4_^3−^) bands (Figure 4), and revealing the formation of AB-type CAp-CB scaffolds. HN analysis determined that the carbonate content of CAp-CB scaffolds was 9.7%, which is close to that of human bone (5–9%) [41,42,43,44].

**Figure 5:**
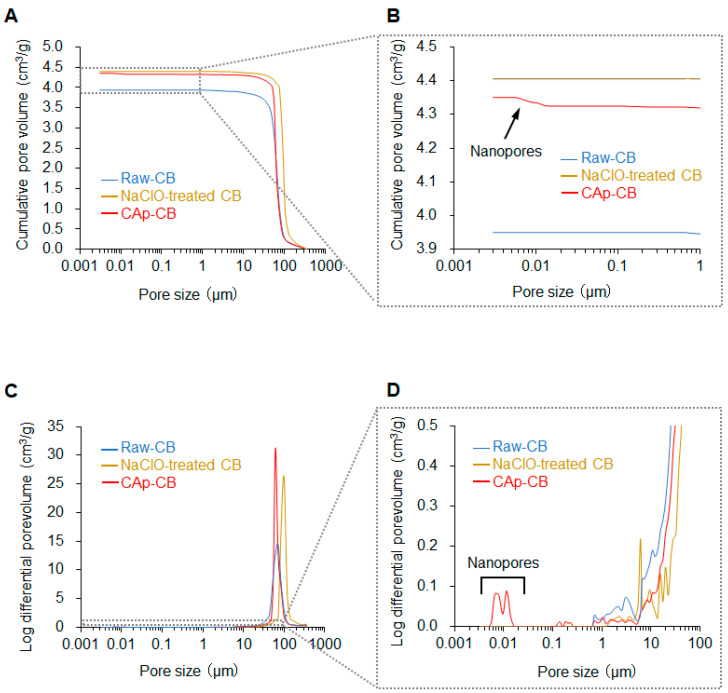
MIP results. (**A**,**B**) Cumulative pore volumes and (**C**,**D**) pore size distributions of raw and NaClO-treated CB cylinders and CAp-CB scaffolds.

The average bulk porosities of raw CB cylinders, NaClO-CB cylinders, and CAp-CB scaffolds were 92.7% ± 0.4, 94.2% ± 0.4, and 93.8% ± 0.6, respectively. The MIP results demonstrate that the total pore volumes of raw and NaClO-treated CB cylinders were 3.95 and 4.40 cm^3^/g, respectively (Figure 5A,B). This increase in the total pore volume resulted from removing organic components from the raw CB cylinders through the NaClO treatment. The total volume of the CAp-CB scaffold was 4.35 cm^3^/g, which is almost equal to that of the NaClO-treated CB cylinders. The macropore size range (40–140 μm) was almost equal between raw and NaClO-treated CB cylinders and CAp-CB scaffolds (Figure 5C,D). Although the raw and NaClO-treated CB cylinders had no nanopores, the CAp-CB scaffolds had nanopores ranging from 0.005 to 0.019 µm (Figure 5C,D). This is because the crystal shape changed from spherical to rod CAp upon phosphatization (Figure 1N,O). The presence of nanopores in the CAp-CB scaffolds may be beneficial for bone regeneration because nanopores have been reported to promote osteoclastogenesis, followed by osteogenesis [45].

### 3.2. Mechanical Properties of CAp-CB Scaffolds

**Figure 6:**
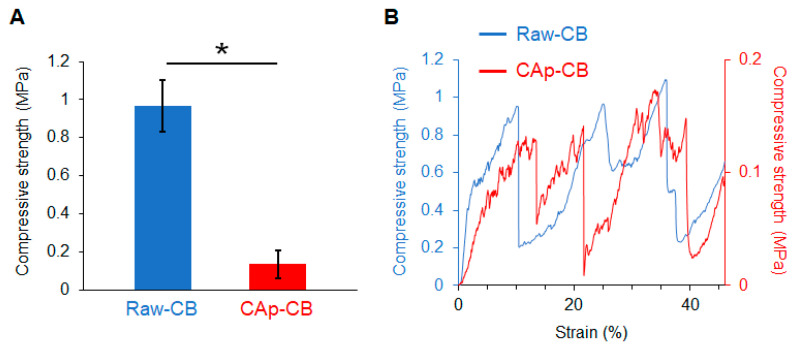
(**A**) Compressive strengths of raw CB and CAp-CB (*n* = 8). * *p* < 0.005. (**B**) Stress–strain curves of raw CB and CAp-CB.

The compressive strengths of raw CB cylinders and CAp-CB scaffolds were 0.96 and 0.13 MPa, respectively (Figure 6A). This decrease in compressive strength was caused by the removal of organic components from the raw CB cylinders. However, despite decreased mechanical strength, the CAp-CB scaffolds could be easily handled without disintegrating, owing to their lamellar septum-like cellular structure. Importantly, layer-by-layer failure behavior, which is an inherent mechanical feature of raw CB, persisted in the CAp-CB scaffolds (Figure 6B). This is because the lamellar septum-like cellular structure that underlies this unique mechanical feature of raw CB was preserved even after the fabrication processes, including organic removal and composition conversion. The layer-by-layer failure behavior may prevent complete failure of the CAp-CB scaffolds when an impact is applied. Although low-impact resistance is a fundamental weakness of ceramics, CAp-CB scaffolds may overcome this weakness due to the layer-by-layer failure behavior endowed by lamellar septum-like cellular structures.

### 3.3. In Vivo Evaluations of Bone Formation

**Figure 7:**
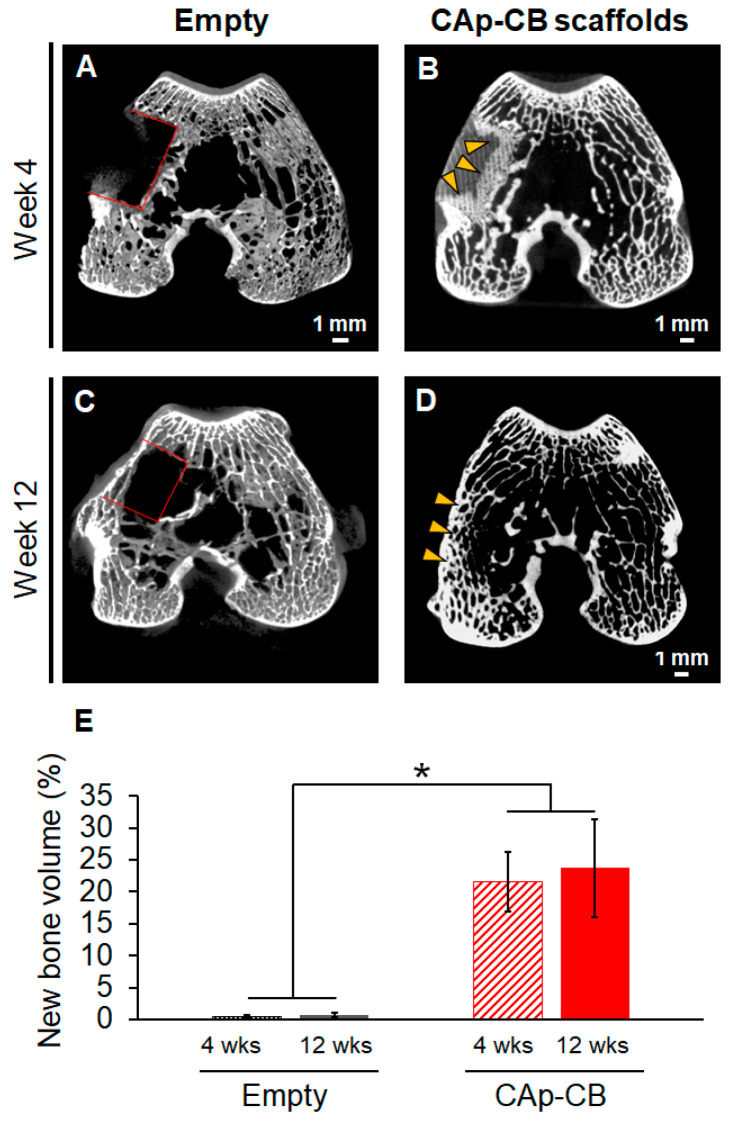
µ-CT images of (**A**) empty and (**B**) CAp-CB scaffold-implanted groups four weeks after surgery. (**C**) Empty and (**D**) CAp-CB scaffold-implanted groups at twelve weeks after surgery. (**E**) Volume percentages of new bone in the defect in the empty and CAp-CB scaffold-implanted groups at four and twelve weeks after surgery. Red lines and yellow arrowheads indicate the bone defect and new bone formed in the defect, respectively. * *p* < 0.005.

µ-CT images showed that four weeks post-implantation, little bone formed in the empty group (Figure 7A). In contrast, in the CAp-CB scaffold-implanted group, new bone grew from the host bone into the scaffold (Figure 7B). A lamellar septum-like cellular structure was visualized in the bone ingrowth region. Thus, the bone penetrated the CAp-CB scaffolds through their macropores, that is, cells enclosed by walls and septa. However, the bone formation in the central region of the CAp-CB scaffold was unclear. Twelve weeks post-surgery, little bone formed in the interior of the bone defect, although the sutured periosteum recovered in the empty group (Figure 7C). In contrast, in the CAp-CB scaffold-implanted group, the scaffold was almost completely resorbed and replaced with new bone (Figure 7D). The new bone formed throughout the bone defect and was continuous with the host bone (Figure 7D). The volume percentages of new bone in the defect in the empty and CAp-CB scaffold-implanted groups were 21.5 ± 4.6 and 0.5 ± 0.2% at four weeks and 23.7 ± 7.5 and 0.6 ± 0.3% at twelve weeks, respectively. The new bone volume in the CAp-CB scaffold-implanted groups at four and twelve weeks was equal to that in the normal bone (20–30%) [46].

**Figure 8:**
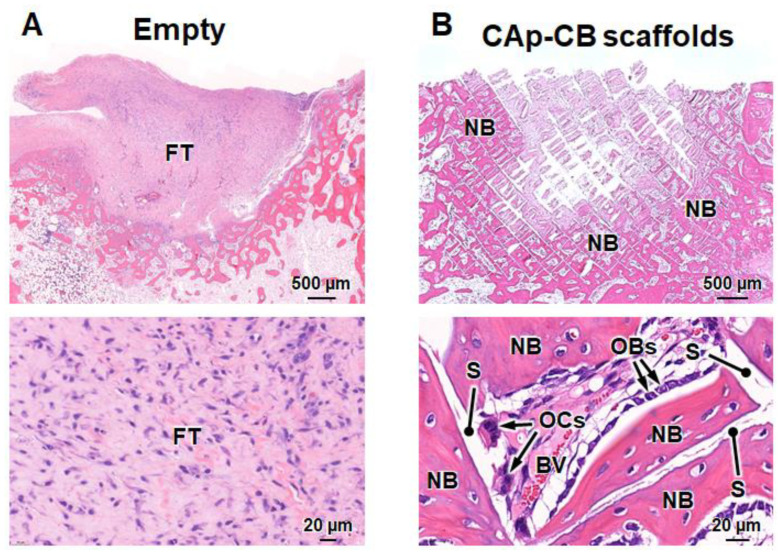
HE-stained sections of (**A**) empty and (**B**) CAp-CB scaffold-implanted groups four weeks post-implantation. (**Upper**) Images showing entire bone defects. (**Lower**) High-magnification images for identifying cells and tissues in the bone defects. “OBs”, “OCs”, “NB”, “BV”, “FT”, and “S” indicate osteoblasts, osteoclasts, new bone, blood vessel, fibrous tissue, and scaffold, respectively.

Histological images clarified that fibrous tissues filled the bone defect four weeks after implantation in the empty group (Figure 8A), which coincides with the results in the same bone defect model [47]. In contrast, in the CAp-CB scaffold-implanted group, new bone was formed in the region outside the center of the scaffold (Figure 8B). New bone was formed within the cells (macropores) of the CAp-CB scaffolds (Figure 8B). These results coincide with the µ-CT result (Figure 7B). Furthermore, the osteoblasts and osteoclasts resided in the new bone and scaffolds, respectively (Figure 8B). The new bone that formed within the scaffold cells (macropores) was continuous with the host bone, and the scaffold in contact with the host bone was resorbed and replaced with bone. These findings demonstrate that the CAp-CB scaffold was resorbed by osteoclastic resorption and was gradually replaced with new bone. Thus, the CAp-CB scaffolds exhibited an in vivo behavior suitable for bone regeneration.

**Figure 9:**
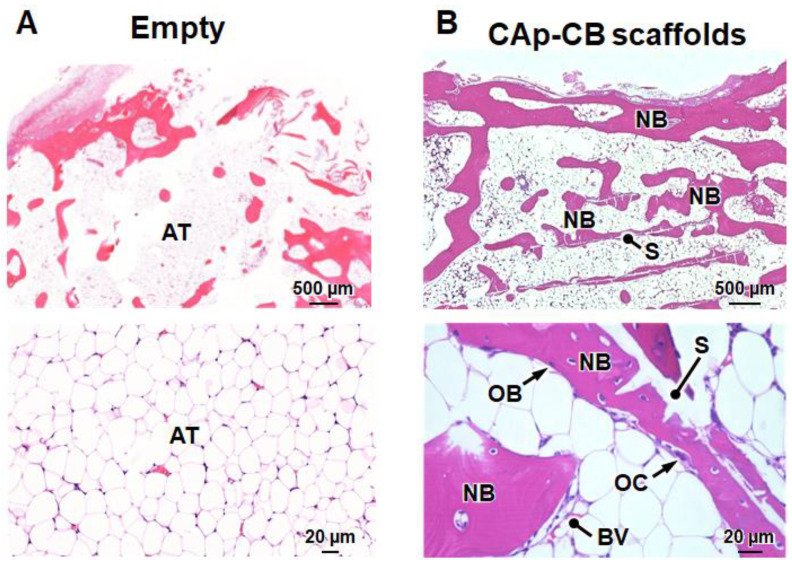
HE-stained sections of (**A**) empty and (**B**) CAp-CB scaffold-implanted groups twelve weeks post-implantation. (**Upper**) Images showing entire bone defects. (**Lower**) High-magnification images for identifying cells and tissues in the bone defects. “OB”, “OC”, “NB”, “BV”, “AT”, and “S” indicate osteoblast, osteoclast, new bone, blood vessel, adipose tissue, and scaffold, respectively.

Twelve weeks after surgery, histological images revealed that adipose tissues occupied the bone defects in the empty group (Figure 9A). In contrast, in the CAp-CB scaffold-implanted group, the scaffolds were almost entirely replaced with new bone, which filled the bone defects (Figure 9B). These results coincided with the µ-CT results (Figure 7D). 

**Figure 10:**
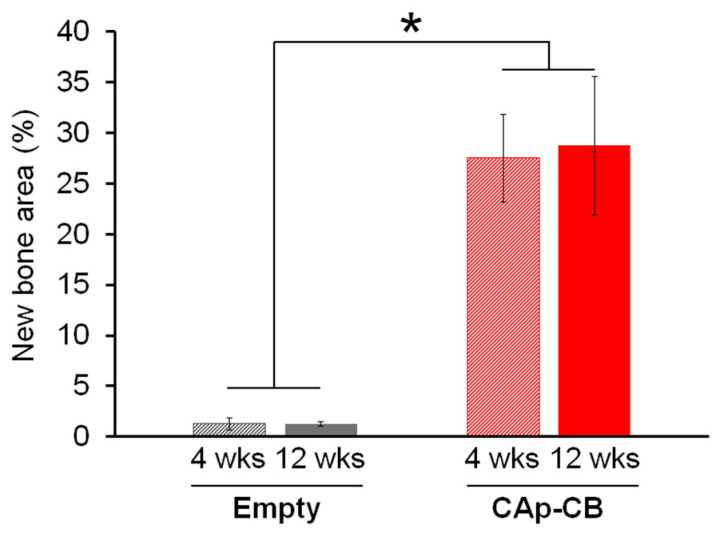
Area percentages of bone newly formed in the defect area of empty and CAp-CB scaffold-implanted groups at four and twelve weeks after surgery. * *p* < 0.005.

The new bone percentages at four weeks after surgery in the empty and CAp-CB scaffold-implanted groups were 1.2 ± 0.5% and 27.5 ± 4.3%, respectively (Figure 10). The new bone percentages at 12 weeks after implantation in the empty and CAp-CB scaffold-implanted groups were 1.2 ± 0.2% and 28.7 ± 6.8%, respectively (Figure 10). The bone percentage in the normal rabbit is reported to be 20–30% [48,49]. Thus, already after four weeks of implantation, the CAp-CB scaffolds generated an amount of new bone equal to the amount of bone in the normal rabbit bone. Furthermore, the CAp-CB scaffolds were almost fully resorbed at 12 weeks after implantation while maintaining the bone amount equal to the amount of bone in the normal rabbit femur.

The conventional fabrication of hydroxyapatite scaffolds derived from CB (HAp-CB scaffold) involves a two-step process comprising hydrothermal treatment followed by sintering [30,31,32]. For example, Rocha et al. reported the fabrication of HAp-CB scaffolds using a hydrothermal treatment at 200 °C followed by sintering at 1100–1400 °C. The obtained HAp-CB scaffolds were densified and lacked micropores through sintering at high temperatures. Consequently, the HAp-CB scaffolds were considered to show poor bioresorbability by osteoclasts. In contrast, the CAp-CB scaffolds underwent little densification because the fabrication process involved no sintering treatment. Furthermore, owing to the crystal shape change from spherical to rod-like through hydrothermal treatment, the CAp-CB scaffolds acquired enhanced roughness and nanopores, which were absent in the HAp-CB scaffolds [32]. In studying alternative organic removal methods for CB, Battistella et al. removed the organics from CB by combining 8% NaClO solution treatment and hydrothermal treatment at 200 °C [50]. However, this hydrothermal treatment at 200 °C caused the carbonate in the apatitic crystal to disappear. Consequently, HAp-CB, and not CAp-CB, scaffolds were yielded. In contrast, our present study removed organics from CB through treatment with a 12% NaClO solution at room temperature, which allowed for the formation of CAp-CB scaffolds with carbonate content similar to human bones. Furthermore, the HAp-CB scaffolds fabricated by Battistella et al. were also densified and lost nanopores, whereas the CAp-CB scaffolds maintained nanopores. The differences in carbonate content and microporosity between the CAp-CB and HAp-CB scaffolds may cause differences in bioresorbability and bone formation.

Additionally, Kim et al. reported bone formation using pure HAp and HAp-CB granules [51]. These granules were implanted into rabbit calvarial defects. The new bone percentages in empty defects, pure HAp-implanted defects, and HAp-CB granule-implanted defects were 3.3, 14.2, and 17.3% at week two after implantation, and 8.6, 20.1, and 25.4% at week eight, respectively. The results from the empty group demonstrated that the rabbit calvaria defect model was easily repaired spontaneously compared to the rabbit condyle defect model in this study. Despite this, the CAp-CB scaffolds formed bone faster and in larger amounts than the pure HAp and HAp-CB granules. Furthermore, Kim et al. demonstrated that a CB-derived lamellar septa-like cellular structure was effective for bone regeneration. The comparison between our study and the study by Kim et al. suggests that CAp is superior to HAp in the context of bone regeneration. This may be due to the lower surface free energy of CAp compared to that of HAp, which promotes protein adsorption and eventually promotes bone formation and maturation [37]. Thus, CAp-CB scaffolds achieved favorable bone regeneration because of their structure and composition.

Moreover, Hayashi et al. reported bone formation in clinically used dense CAp granules of different sizes using the same bone defect model as that used in this study [26,46,47,52]. At week four after implantation, the bone percentages in the defects implanted with clinically used dense Cap granules that were 1–2, 0.6–1, and 0.3–0.6 mm in size were approximately 12% [45,52], 20% [26], and 21% [46], respectively. Thus, CAp-CB scaffolds can regenerate bone faster than clinically used dense CAp granules. Furthermore, Hayashi et al. demonstrated that scaffolds with macropores, such as honeycomb, chain, and gear-shaped scaffolds, promoted bone regeneration better than clinically used dense CAp granules [26,46,47,52]. These findings indicate that the lamellar septa-like cellular structure of the CAp-CB scaffold is as effective as the honeycomb, chain, and gear structures for bone regeneration.

These findings indicate that CAp-CB scaffolds promote bone regeneration better than clinically used scaffolds. However, the scaffold structure can be improved to promote further bone regeneration. Hadagalli et al. improved cell attachment to HAp scaffolds by controlling the porous structure through sintering a mixture of CB-derived HAp powder and organic pore formers such as wax, wheat flour, and milk powder [53]. Although the lamellar septa-like cellular structure of CB disappeared in the scaffolds fabricated using this method, improvements in this method may promote bone regeneration while preserving the lamellar septa-like cellular structure.

## 4. Conclusions

We successfully fabricated lamellar septa-like cellular-structured CAp scaffolds (CAp-CB) by removing the organic components of CB through NaClO treatment and the subsequent HT of the NaClO-treated CB. The CAp-CB scaffolds exhibited layer-by-layer fracture behavior, which is a unique mechanical property of CB. Although the CAp-CB scaffolds possessed 93% porosity, they were easily manipulated and implanted into rabbit femoral bone defects. Four weeks after implantation, bone formed in the cells, i.e., macropores, of the CAp-CB scaffolds. Twelve weeks after implantation, the CAp-CB scaffolds were almost replaced with new bone, which filled the bone defects completely. Based on these findings, CAp-CB scaffolds may not be completely destroyed by compressive impact during bone regeneration owing to their layer-by-layer fracture behavior, which may allow them to continue to serve as scaffolds for bone regeneration.

## Data Availability

The datasets supporting the findings of this study are available from the corresponding author upon reasonable request.
